# Existential aspects documented in older people’s patient records in the context of specialized palliative care: a retrospective review

**DOI:** 10.1186/s12913-022-08753-1

**Published:** 2022-11-16

**Authors:** Marina Sjöberg, Birgit H. Rasmussen, Anna-Karin Edberg, Ingela Beck

**Affiliations:** 1 Department of Care Science, Faculty of Health and Society, Malmö University, Malmö, Sweden; 2 The Research Platform for Collaboration for Health, Faculty of Health Sciences, Kristianstad University, Kristianstad, Sweden; 3 The Institute for Palliative Care, Lund University and Region Skane, Lund, Sweden; 4 Department of Health Sciences, Faculty of Medicine, Lund University, Lund, Sweden; 5 Department of Clinical Sciences, Faculty of Medicine, Lund University, Lund, Sweden

**Keywords:** Documentation, Existential aspects, Palliative care, Frail elderly, End of life

## Abstract

**Background:**

Documentation of older people’s end-of-life care should cover the care given and provide an overview of their entire situation. Older people approaching the end of life often have complex symptoms, live with bodily losses, and face an unknown future in which existential aspects come to the forefront. Knowledge of the existential aspects recorded in palliative care documentation is sparse and merits improvement. This knowledge is relevant to the development of more holistic documentation and is necessary in order to promote reflection on and discussion of documentation of the sensitive existential considerations arising in palliative care. The aim of this study was to describe the documentation of existential aspects in the patient records of older people receiving specialized palliative care.

**Methods:**

Data were obtained from a retrospective review of the free-text notes in 84 records of randomly selected patients aged ≥75 years enrolled in specialized palliative care units who died in 2017. The notes were analysed using an inductive qualitative content analysis.

**Results:**

The notes documented existential aspects in terms of connotations of well-being and ill-being. Documented existential aspects were related to the patients’ *autonomy* concerning loss of freedom and self-determination, *social connectedness* concerning loneliness and communion, *emotional state* concerning anxiety and inner peace, and *state of being* concerning despair and hope. The notes on existential aspects were, however, not recorded in a structured way and no care plans related to existential aspects were found.

**Conclusions:**

Existential aspects concerning both ill-being and well-being were sparsely and unsystematically documented in older people’s patient records, but when notes were extracted from these records and analysed, patterns became evident. Existential aspects form an important basis for delivering person-centred palliative care. There is a need to develop structured documentation concerning existential aspects; otherwise, patients’ thoughts and concerns may remain unknown to healthcare professionals.

## Background

Clinical record-keeping is integral to delivering safe, high-quality healthcare and is fundamental to clinical practice [1, 2]. WHO guidelines emphasize that documentation in patient records should be person centred, include objective and subjective data used in assessing patient needs and health status, be individualized, and lead to a current care plan [2]. Documentation constitutes a basis for communication between healthcare professionals (HCPs) and patients [3]; it should provide a comprehensive overview of the patient’s situation and of the interventions planned with the patient to ensure safe, high-quality care [4].

### Existential aspects of older people at the end of life

The literature describes existential aspects encountered towards the end of life in terms of both suffering [5] and well-being, concepts closely related to health and spirituality [6]. Experiences of existential well-being/health/spirituality include: feelings of connectedness to the self, others, and/or nature or a higher power; a sense of meaning in life; and a sense of transcendence beyond the self in everyday living [6, 7]. Existential suffering is described as including experiences of loss of personal meaning, loss of purpose in life, fear of death, anxiety, hopelessness, fear of burdening others, loss of dignity, and loneliness [5]. Frail older people approaching the end of life often have complex health problems and an increased vulnerability to stressors [8]. For most people close to the end of life, existential issues come to the fore [9]. When facing death, various existential aspects can emerge and cause suffering [5, 10]. Specifically among older people, deteriorating body functions can affect and trigger a sense of existential loneliness, i.e., a deep feeling of loneliness [11]. Furthermore, among people with physical multimorbidity, loneliness has been shown to increase the risk of developing several other illness-related issues, such as depression and anxiety [12].

Research shows that living towards the end-of-life entails facing loss and adapting to a new and unknown existence in which the person strives for balance by calibrating and adjusting expectations in order to maintain physiological and existential well-being [13, 14]. This might require support from other people, such as family members and HCPs, to promote quality of life and help the older person prepare for approaching death [15, 16]. As factors such as culture, financial issues, religion, age, disease, and life circumstances all shape individuals, end-of-life care must be individualized and person centred [17]. Giving older people the opportunity to express their wishes, thoughts, concerns, beliefs, and values is important for their quality of life [18–20], and thus their existential health. A dying person’s preferences, beliefs, and values constitute important information to pass on to the team of HCPs caring for that person and his/her family, as this information has been shown to bolster patients’ experiences of safety and care continuity [21]. Such knowledge is also essential for HCPs’ ability to plan for and provide high-quality end-of-life care.

### Documentation of existential aspects in patient records

Advanced care plans, which are prepared by both non-palliative-care–trained HCPs and specialized palliative nurses, are documents covering the physical, social, psychological, and spiritual domains and stating individuals’ goals and preferences for future treatment and care. A study of such plans in specialized palliative care found that they were most concerned with the physical domain, much less with the social and psychological domain, and least with the existential domain [22]. This is in line with a study of documentation in end-of-life care in nursing homes, which found that HCPs mainly focused on physical symptoms and that existential issues were almost completely unmentioned in the records of residents affected by dementia [23]. In a study of specialized palliative care from 2008, Gunhardsson et al. [24] noted that documentation of physical problems and needs (especially pain) was most common, and that notes about existential/spiritual care were sparse, fragmented, and unorganized. We obtained similar results in a recent study from 2021, examining the content of documentation in older people’s patient records in a specialized palliative care context, finding a high frequency of documentation of physical problems such as pain and of interventions related to pharmacological treatment [25]. Albeit to a lesser and sporadic extent, the free-text notes in the documentation covered patient wishes, well-being, and aspects of dying. It is, however, a premise for the delivery of safe, person-centred palliative care that psychological, social, and existential aspects as well as the interventions planned with the patient should be documented as well. In this paper, the free-text documentation is the focus of more in-depth analysis.

### Aim

The aim of this study was to describe the documentation of existential aspects in the patient records of older people who had received specialized palliative care.

## Methods

### Study design

This study was a retrospective review of free-text notes in patient records, analysed using inductive content analysis as described by Elo and Kyngäs [26]. The data were collected in connection with a previously conducted study of the documentation in older patients’ records in specialized palliative care [25]. The present study is part of the LONE project [27], examining existential loneliness among frail older people.

### Sample

The sample in this study was derived from 11 palliative units providing specialized palliative care in three geographical areas in southern Sweden. The inclusion criteria were patient records of people aged > 75 years who had received end-of-life care in an in-patient ward and/or in their own home and had died between 1 January and 31 December 2017. Every eighth patient record meeting the criteria was randomly selected in each of the three geographical areas, resulting in 92 electronic patient records. The decision to select every eighth medical record of older people who died in 2017 (*n* = approximately 750) [28] was based on the sample for the previously conducted quantitative study [25]; it was estimated that at least 80 medical records were needed in that study. Of these 92 patient records, eight patient records did not include any free-text notes about existential aspects and were excluded. This resulted in a total sample of 84 patient records. Of the remaining 84 patient records, 35 (42%) were for women and 49 (58%) for men. The mean patient age was 83 years (range 75–94) and the time in palliative care was two–312 days with a mean of 59 days (median 28). The most common diagnosis was cancer (85%); other diagnoses were lung diseases (not cancer), neurological diseases (e.g., ALS and Parkinson’s disease), and multiple diagnoses. For a more detailed description of the patients, see Table 1.

**Table 1:** Description of the sample

Patient record	*n*=84
Men/women	49 (58%) /35 (42%)
Age, median (range)	83 years (75–94)
Enrolled in palliative care (mean/median)	2-312 days (59/28)
Lived alone/ lived with family	38/46
Cared at own home/ at palliative in-patient ward	24 (29%)/ 32 (38%)
Combination	28 (33%)
Died in own home/ in a palliative in-patient ward	24/ 55
Died alone	16
Own home/ in-patient ward	2 (7%)/ 14 (27%)
Significant others	
Spouses and children	73
Other	10
Nobody	1

### Setting

In terms of total capacity, the 11 palliative units serve patients of all ages, providing palliative care for approximately 280 patients at home and 70 in palliative in-patient wards. About 1650 patients die in these specialized palliative care units yearly, of whom about 54% are aged ≥75 years, representing approximately 750 deceased people in 2017 [28]. Older people enrolled in specialized palliative homecare and in-patient wards have complex needs, and diagnoses such as cancer, lung and heart diseases, neurological diseases, and multi-morbidity are common. In total, the studied units have approximately 450 employees: physicians (8%), registered nurses (59%), and other professionals (32%). The units’ multidisciplinary teams consist of physicians (leading the teams), registered nurses, and licensed practical nurses (working in in-patients ward only) and are available around the clock. Professions such as physical therapists, occupational therapists, dieticians, and social workers are available during the day.

### Data collection

Data were collected by the first author by identifying the free-text notes concerning existential aspects in the patients’ records. A random selection of about 5% of the extracted data was independently reviewed by two of the four co-authors (BHR and IB). As others have noted, the concepts “spiritual” and “existential” are used interchangeably in the literature; with no single commonly accepted definition of these concepts (see, e.g., [29–31]), we used the concept “existential” in its broadest sense. Data consisted of free-text notes in relation to: (1) the patient’s wishes and priorities, i.e., notes about the patient’s wishes or about related aspects of importance; (2) problems, symptoms, and needs; (3) strategies, i.e., notes about how the patient handles her/his situation; (4) wellbeing, i.e., notes about the patient’s strengths and sense of emotional balance, meaning, and community; (5) communication/conversation; (6) presence and touch; and (7) interventions. A total of 22,640 clinical notes were identified in the previous study (25); of these, 2088 free-text notes (approximately 9%) addressing existential aspects were identified in the patients’ records.

### Analysis

From the patient records, the free-text notes concerning existential aspects were analysed using qualitative content analysis, in a method incorporating the phases of preparation, organization, and categorization [26]. The preparation phase started with reading the free-text units, which were entered into an analysis matrix. All four authors independently read the data in the matrix several times and then came together to discuss the content. The next step was condensing the notes and organizing them under headings on a coding sheet. This step was carried out by the first and last authors. All headings were then grouped based on how they related to one another and were assigned higher-order headings. In the categorization phase, four categories were created that describe the documentation of existential aspects in the patient records (Table 2). In each of the four categories, the notes were, according to their connotations, sorted into elements of either well-being or ill-being. The intention was to stay close to the text during the analysis. The interpretation of the notes was guided by the context and the situation described in the notes. For example, if a note said, “hoped to fall asleep and not wake up”, the note was interpreted considering the documentation of the patient’s situation as a whole as concerning either well-being or ill-being, and in this case was sorted under the category of well-being. When using the concept of “notes”, we are referring to the individual notes in the patient records; these are presented in quotation marks in the text and are derived from the patient records and not from the patients themselves. Throughout the data analysis process, all four authors met to discuss the analysis, and any discrepancies were reflected on and discussed until consensus was reached.

**Table 2:** The four categories of existential aspects documented in the patient records

Categories	
Aspects related to personal autonomy	
Aspects related to social connectedness	
Aspects related to emotional state	
Aspects related to state of being	

## Findings

The notes understood as treating existential aspects conveyed connotations of both ill-being and well-being and were represented in all four categories (Table 2). An incidental finding was that the free-text notes describing existential aspects in the patient records were usually intermixed with other information and were not structured according to specific keywords associated with existential aspects. Furthermore, no care plans specifically addressing existential aspects were found.

### Aspects related to personal autonomy

The notes mentioned existential aspects related to personal autonomy, concerning ill-being in terms of *loss of freedom* and wellbeing in terms of *self-determination*.

Notes relating to *loss of freedom* expressed the hardship of not being able to move around or take care of oneself and of having to depend on others for help. The notes described feelings of frustration at not being able to do things one could do in the past: “Is not afraid of death, but thinks it is hard that she now needs help as she has always managed by herself” (note about a woman, 91 years old, in-patient ward).

The notes described situations in which lack of energy hindered the older people from doing what they wanted to do, and some notes described a process of coming to accept the present reality. The state of being tired and powerless made the patients sad. Other notes referred to the experience of feeling unsafe going outdoors, due to lack of bodily energy; this meant that patients did not dare to go outside, instead staying at home and becoming isolated. Notes also described feelings of frustration expressed, for example, when the older people were not informed of their condition, were “asked to pee in a diaper”, or met other people who did not understand their current life situation.

The notes concerning *self-determination* recorded the patients’ desire to take care of themselves as much as possible, for example, taking prescribed medications themselves and contacting municipal homecare services by themselves to discuss the kind of help they needed. Expressions such as “wants to manage as much as possible”, “wants to ask for help themselves”, and “wants control” were found in the notes. Although the patient’s body was gradually deteriorating and weakening, the notes described a patient as “using the aids available to make everyday life easier and to save energy”. Other notes stated that patients preferred not to talk about the future and imminent death at all, or that an older person had “learned to push away emotions and move on”. Although notes described one older person as preferring not to talk about death, several notes stated that s/he was aware that the end of life was near. The notes revealed that the patient wished to be involved in her/his own care, for example, by participating in care decisions and being able to refuse offered care. The notes also articulated the importance for the patients of deciding on the physical care of their bodies. The notes stated that it was important for all HCPs to know their patients’ individual desires when, in the future, they were no longer able to express their wishes.

### Aspects related to social connectedness

The notes mentioned existential aspects related to social connectedness, specifically ill-being in terms of *loneliness* and wellbeing in terms of *communion.*

The documentation of the older people’s ongoing lives contained notes about feelings of *loneliness*, expressed in the records in various ways. For example, notes mentioned patients not wanting to be alone, wanting to have people around, wanting to move to a nursing home because of a lack of social interaction, and feeling lonely even when with other people, revealing feelings of emptiness and boredom. Furthermore, notes described how an older person simply lay down waiting for the next visit, while other notes described an older person’s longing for company during meals. The following note recorded a patient’s feeling of loneliness and desire for someone to be nearby: “Is feeling worried after her son left, feeling lonely and wanting someone to check on her regularly” (woman, 91 years old, in-patient ward).

Notes also revealed that the patients were missing significant others who had died. Other notes described an older person who gripped hold of the HCPs so as not to be left alone. Furthermore, notes described patients who, despite expressing feelings of loneliness, rejected invitations to sit in the living room with other people.

Notes identified as expressing *communion* related to older people being with their family and HCPs in their own homes or in the palliative in-patient wards. These notes described activities such as talking, being together, and celebrating events as well as the patients’ moods, for example: “was in a good mood” and “was laughing and joking”. In some notes, HCPs described conversations in which the older people were allowed to take their time and talk about whatever they wanted. Other notes indicated that they spent time communicating with their children via Skype and spent time with their pets, even in the in-patient ward, which was described as making them happy and satisfied. The following quotation from a note recorded the type of activity and mood described by an older person who had celebrated a baptism: “Attended a baptism on the weekend and endured participation in church. Feels satisfied by that” (woman, 90 years old, own home).

The notes in this category also spoke of the older people as receiving good support and help from family, describing the family and/or HCPs as being present and watching at their deathbeds.

### Aspects related to emotional state

The notes mentioned existential aspects related to emotions, concerning ill-being in terms of *anxiety* and wellbeing in terms of *being at peace.*

The notes related to *anxiety* were always followed by explanations as to why such feelings were expressed. The anxiety could concern being worried and stressed about having to move to a nursing home or could be because other patients were straying into their room in the in-patient ward. Notes also described the patients’ experiences of unpleasant symptoms, for example, fears about “not getting enough air” and that the problem could worsen in the future. The notes about anxiety also concerned fear of dying. Documented feelings of anxiety were often related to nightly events such as nightmares and sleeplessness caused by intrusive thoughts: “The patient is very anxious, is afraid to fall asleep due to fear of not waking up” (man, 79 years old, own home).

Feelings of anxiety in the present were often caused by thoughts about the past or future, rather than about the situation here and now. The notes revealed the patients’ worries about what would happen to their families when they were dead. Notes also described anxiety about a future of anticipated pain, severe symptoms, and insufficient help. The notes described interventions such as HCPs offering contact with other professions, usually social workers and physicians, and/or contact with religious representatives for further conversations. The notes reported conversations with the older people in which suggestions were offered about, for example, how to relieve anxiety by soft-touch massage. Other interventions for calming anxious patients were “getting tucked in”, “getting help to listen to music”, and “sitting beside a dying person”.

Notes related to *being at peace* were varied and referred to the past, present, and expected future life. Notes describing the present life told of “coming to peace”, “sleeping well”, “having a good time”, “feeling well”, and “being in good spirits”. Further notes contained information about the patients “accepting their situation, wanting to take advantage of the day and do nice things with their family”. Notes related to the past life could describe a patient as “being satisfied with the life lived” and, in relation to the future, as “not feeling any fear or anxiety about having to die soon” because s/he “has planned and arranged for their family and know they will be safe”. Even though the patients felt safe and peaceful, the notes documented their wishes for support from HCPs: “Is not anxious about what’s ahead but wants support at the end of life” (woman, 81 years old, own home).

### Aspects related to state of being

In the notes, existential aspects related to state of being were found, and concerned ill-being in terms of *despair* and wellbeing in terms of *hope.*

The notes related to *despair* revealed that the dying older people were living with severe problems. These notes often recorded the words of the patients who felt that life was not worth living. Their present life was described as a “meaningless existence”, associated with the impossibility of living the way they wished to live. Notes also recorded an increased sense of boredom, sadness, and tiredness, making it difficult to find joy or any quality of life, leading to a desire to end one’s life. The notes about patients wanting to die or to shorten their lives were worded in various ways: “unable to live”, “does not want to live any longer”, “wishes for euthanasia”, and “crying and expressing a desire to shorten life”. Some notes described older people “becoming panicky” or screaming “I want to die”, but went on to report that they eventually calmed down.

The notes related to *hope* reported patients’ wishes to live for a while longer, even though they were aware that death was imminent. Other notes described the hopefulness of older people striving to live in the present and “take the days as they come”, “focus on the present”, and “take advantage of the times of well-being”. Other notes focused on the future and expressed patients’ hopes of being able to celebrate upcoming birthdays, and expressions such as “hopes to get better” and “wants to fight on”, and reports of patients’ wishes to continue life-prolonging treatments were found. Notes described how the patients wished to go outdoors and “looked forward to sitting in a wheelchair” or “longed for the spring” or “to get home and be able to go outside in their garden” when in the in-patient ward. Some notes described patients’ feelings of joy experienced between periods of suffering. Furthermore, the notes also revealed hopes of letting go of life. The notes described patients’ wishes for a death that “will be fast and be painless” and that they will be supported by HCPs and not left alone. One note described a patient’s hope “to fall asleep and never wake up again”.

## Discussion

The results indicate that the documentation of existential aspects in older people’s patient records within specialized palliative care covered elements of both ill-being and well-being. Yet, the notes were sparse and unsystematic, were intermixed with other kinds of documentation, could not be identified by specific keywords, and did not result in any documented care plan shared with the patients. However, when the notes were extracted and analyzed, patterns of well-being and ill-being became evident. The existential aspects related to ill-being were loss of freedom, loneliness, anxiety, and despair and could be described as existential suffering or its causes [5]. The existential aspects of well-being, on the other hand, related to self-determination, communion, being at peace, and hope and could be described as existential health [7, 17]. A review of existential suffering among patients in palliative care settings found several definitions of existential suffering, including descriptions of loss of personal meaning, loss of purpose in life, fear of death, anxiety, hopelessness, fear of being a burden to others, loss of dignity, and loneliness [5]. These descriptions of existential suffering were identified in our study as elements of ill-being. Furthermore, Boston et al. [5] found fundamental themes that were pervasive in the care of the dying – i.e., life and death, hope and despair, and relationships and isolation – themes that recall the present findings. However, the notes indicated that descriptions of a deteriorating body were also fundamental and, in the documentation, closely connected to notes on loss of freedom when one is dependent on others. In another study conducted in nursing homes, the body was in focus when older residents were talking about death and dying, which were often linked to their bodily experiences [32]. Studies have found that living in a deteriorating body is a prominent experience triggering a sense of “homelessness” [33] and existential loneliness among dying patients with cancer [9] and among frail older people [11]; a sense of being homeless in life was related to loneliness, which was further described as existential suffering [34]. Existential suffering was closely related to no longer being the person one wants to be in the eyes of others, resulting in a loss of one’s dignity and identity [35]. In contrast to loss of freedom and being dependent on others, some notes concerned self-determination, expressing the older people’s wishes to take care of themselves as much as possible, participate in decisions about their care, and be able to refuse the care offered. It thus seems important to consider not only aspects of loss, but also aspects that can strengthen the experience of dignity, despite a deteriorating body.

Our study identified some notes indicating supportive care interventions related to the older person’s ill-being as well as well-being, interventions such as “tucking in” the patient, giving soft-touch massage, helping the patient listen to music, having conversations, and sitting beside the dying person to relieve her/his anxiety. The notes also indicated that these interventions were experienced as supportive, as the patients were described, for example, as “sleeping well”, “having a good time”, and “laughing and joking”. Several other studies show that supportive care interventions relieve existential suffering [5, 36]. Compassion, an emotional response to the suffering of others, includes engagement and seeking to help those who suffer [37]. This could entail sitting down and listening [37, 38], for example, inviting patients to discuss issues that matter most to them and helping them to formulate how they want to be remembered. According to Chochinov [39], the opportunity to discuss meaningful issues results in a heightened sense of dignity, purpose, and meaning in life as well as reducing psychosocial and existential distress at the end of life. Giving older people at the end of life the opportunity to express their wishes, thoughts, concerns, beliefs, and values has been shown to be important for their quality of life [18–20], but is largely ignored in studies of the content of patient records of end-of-life care [22–25]. In our study, some of these supportive interventions were found, but they were unstructured and sporadic. Research shows that nurses focus on doing rather than being [40] and are insecure and find it difficult to address existential issues [41]. Educating and training HCPs has been shown to be one way to strengthen and empower them to meet older people’s existential needs at the end of life [37].

Documentation of existential aspects in patient records is a sensitive matter, as it records personal wishes, moods, and thoughts that older people might not want to share with all HCPs. This ethical dilemma must be considered when information on existential aspects is documented in patient records and could be a reason why notes on existential aspects are sparse in patients’ records [23, 24]. On the other hand, such information could be valuable for HCPs, making them more aware of patient needs in order to better provide support. Browall et al. [42] found that HCPs believe that they are responsible for paying attention to existential aspects, but that a major obstacle to dialogue about existential aspects was the lack of documented notes. For HCPs, this could lead to uncertainty about how, when, and whether existential aspects have been highlighted and supported. Traditionally, nurses have communicated information about their patients orally and, compared with written information, more information about care planning has been conveyed in such oral communication [43]. However, Ekman et al. [17] described partnership formation and documentation in person-centred care as comprising three steps: listening to the patient’s narrative about his/her own preferences, beliefs, and values is the first step in establishing a partnership with the patient; the next step is shared decision-making building on the partnership; and the last step is to safeguard the partnership by documenting the agreement. This process illustrates open collaboration that legitimizes the information shared with HCPs, which could be included in a care plan. The shared decision-making about existential aspects is important and could be one way to deal with the ethical dilemma concerning the documentation of sensitive information to improve ongoing patient care. However, the sort of structured care plans needed, possible, and helpful for HCPs in addressing existential issues is unknown. Future research should consider to what extent documentation accurately reflects conversations with patients, but also the kind of information acceptable for patients and the sort of structured care plans that will inform the care provided to patients at the end of life. Including the kind of key words beneficial for highlighting existential aspects.

### Methodological considerations

The strengths of this study are that it was conducted in a context in which HCPs are supposed to pay attention to existential aspects, and that the patient records were randomly selected and include variation concerning age, gender, place of care, and diagnosis. In line with our pre-understanding, all authors were involved in the analytical process and continued discussion until consensus was reached, increasing the credibility of the results [44]. Regarding transferability, the random sample was selected from different geographical areas in the same context, as thoroughly described. The presentation of the findings contains several quotations from the studied documentation that, on one hand, bolster the findings’ authenticity [45] but, on the other, do not convey the patients’ own descriptions (which would yield in-depth knowledge) but rather the HCPs’ perceptions of what the patients have expressed. The purpose of this study was, however, to describe what was documented and not what the patients actually expressed.

## Conclusion

Existential aspects concerning both ill-being and well-being were sparsely and unsystematic documented in older people’s patient records, but when the notes were extracted and analysed, patterns became evident. The extracted notes described both ill-being and well-being and revealed that HCPs do document older people’s existential suffering and existential health. Alleviating existential distress should be a care priority when frail older people reach the end of life; this presupposes open collaboration between the older people and HCPs, leading to a shared care plan. Although HCPs do document existential aspects in older people’s patient records, it is unknown how well these notes help them deliver high-quality care to individual older people. There is a need to feature existential aspects in patient records to ensure holistic care at the end of life. Future research should consider to what extent the documentation of existential aspects in patient records accurately reflects conversations on these matters and informs the care provided to older people in specialized palliative care.

## Data Availability

The datasets used and/or analysed during the current study are available from the corresponding author on reasonable request.
